# Rolling epidemic of Legionnaires’ disease outbreaks in small geographic areas

**DOI:** 10.1038/s41426-018-0051-z

**Published:** 2018-03-21

**Authors:** C. Raina MacIntyre, Amalie Dyda, Chau Minh Bui, Abrar Ahmad Chughtai

**Affiliations:** 1 School of Public Health and Community Medicine, Faculty of Medicine, University of New South Wales, Sydney, NSW 2052 Australia; 2 College of Public Service & Community Solutions, Arizona State University, Tempe, AZ 85004 USA

## Abstract

Legionnaires’ disease (LD) is reported from many parts of the world, mostly linked to drinking water sources or cooling towers. We reviewed two unusual rolling outbreaks in Sydney and New York, each clustered in time and space. Data on these outbreaks were collected from public sources and compared to previous outbreaks in Australia and the US. While recurrent outbreaks of LD over time linked to an identified single source have been described, multiple unrelated outbreaks clustered in time and geography have not been previously described. We describe unusual geographic and temporal clustering of *Legionella* outbreaks in two cities, each of which experienced multiple different outbreaks within a small geographic area and within a short timeframe. The explanation for this temporal and spatial clustering of LD outbreaks in two cities is not clear, but climate variation and deteriorating water sanitation are two possible explanations. There is a need to critically analyse LD outbreaks and better understand changing trends to effectively prevent disease.

## Introduction

Legionnaires’ disease (LD) is a cause of community-acquired atypical pneumonia, with sporadic cases and outbreaks reported from many part of the world. The disease is caused by *Legionella* bacterium, which was first detected during the investigation of a major pneumonia outbreak in a convention in Philadelphia in 1976^1^. The primary reservoir of *Legionella* is water supplies, decorative fountains and cooling towers in buildings^2^. The majority of human infections (70–90%) have been caused by *L. pneumophila*, mainly serogroups 1 and 6. Most cases in humans are asymptomatic or cause a mild illness called Pontiac fever, and complications are rare^3^. Symptomatic and severe disease tends to affect older people, especially those with chronic diseases, and males.

*Legionella* is transmitted to humans through inhalation of contaminated aerosols–common environmental sources of these aerosols include drinking water, whirlpool spas, cooling towers and decorative fountains^4^. Other reported sources include humidifiers and produce misters^4^. Human to human transmission generally does not occur but was reported in a LD outbreak in Portugal^5^. The outbreak started on November 2014 and 334 confirmed cases were reported within 1 month^5^. The source of the outbreak was a wet cooling system, but person to person transmission was reported in one case^6^.

Although LD is a notifiable disease in many countries^7^, the disease burden may be underestimated due to a lack of surveillance and routine testing of bacteria in most parts of the world. Limited data are available but the reported incidence is around 10–15 cases detected per million population. Of the reported cases, 75–80% are aged >50 years, 60–70% are male and most cases have underlying chronic diseases^4^. The number of reported cases has been rising in the United States (US), Europe and other developed countries over the past decade^8^. In the US, each year 8000–18,000 people are hospitalized with LD^9^ and the number of reported cases has increased four-fold from 2000 to 2014^10^. This increase in the number of LD cases may reflect a true increase in the frequency of disease due to a number of factors (e.g., ageing populations, more at-risk individuals, ageing plumbing infrastructure and climate variation). However, increased ascertainment may be occurring due to more frequent diagnostic testing, enhanced reporting^11^ and changes to the case definition since 1996^7^. The serological cut-off limit was updated (four-fold increase equal to 1:128 in 1996 to only a four-fold increase in 2005) and travel-associated LD cases were also included^7^.

In most cases, LD outbreaks are discrete and limited to one geographical area. As per the Centres for Disease Control and Prevention (CDC) definition, an outbreak of *Legionella* occurs when 'two or more people are exposed to *Legionella* and get sick in the same place at about the same time'^10^. From 2014 to 2016, multiple outbreaks of LD clustered in time and space were reported from the Bronx, New York City (NYC) and Sydney, Australia. While multiple outbreaks linked to a single source but occurring serially at different times have been described^12,13^, rolling outbreaks associated with different exposures clustered in time and space have not been commonly reported in the past. The aim of this case study is to examine the characteristics of rolling outbreaks clustered in time and space in two cities: Sydney, Australia and New York, US.

## Materials and methods

We collated data on LD outbreaks in NYC and Sydney through searches of relevant published literature in Medline and Embase databases, news reports and websites of the health departments of NYC and New South Wales (NSW). The review was carried out in 2015–2016 and updated in 2017. A step-wise search protocol was used to search Medline and Embase databases, starting initially with MeSH (Medical Subject Heading) terms, coupled with Boolean logical operations. Results of the search were exported to EndNote, and irrelevant sources were removed through a review of title and abstracts. News reports, websites and published literature were analysed to extract information on LD cases and epidemiological investigations, specifically characteristics of cases (age, sex, comorbidities, hospitalization rates), disease onset dates, locations of exposure, potential sources of infection, results of environmental testing for *Legionella* bacteria and public health interventions (such as cleaning of cooling towers).

The CDC collects and reports three types of data on LD outbreaks: (i) surveillance for waterborne disease and outbreaks associated with drinking water, (ii) surveillance for waterborne disease and outbreaks associated with water not intended for drinking, and (iii) surveillance for waterborne disease and outbreaks associated with recreational water use.

Graphs were created using R (version 3.1.3). Base map data was taken from the Australian Standard Geographical Classification (ASGC) Digital Boundaries, Australia^14^. Coordinates of outbreak locations were retrieved by searching names of locations on Google maps. Maps were created using ArcMap 10.2. Disease data for plots and maps were obtained from publicly available sources as described above.

The epidemiologic characteristics of temporally and geographically clustered outbreaks in Sydney and NYC were compared to other previous LD epidemics in the same regions. We specifically compared the total number of LD notifications from previous years, sources of infection and evidence of clusters in both settings

## Results

### Case study 1—multiple LD outbreaks in the Bronx, New York City

**Fig. 1:**
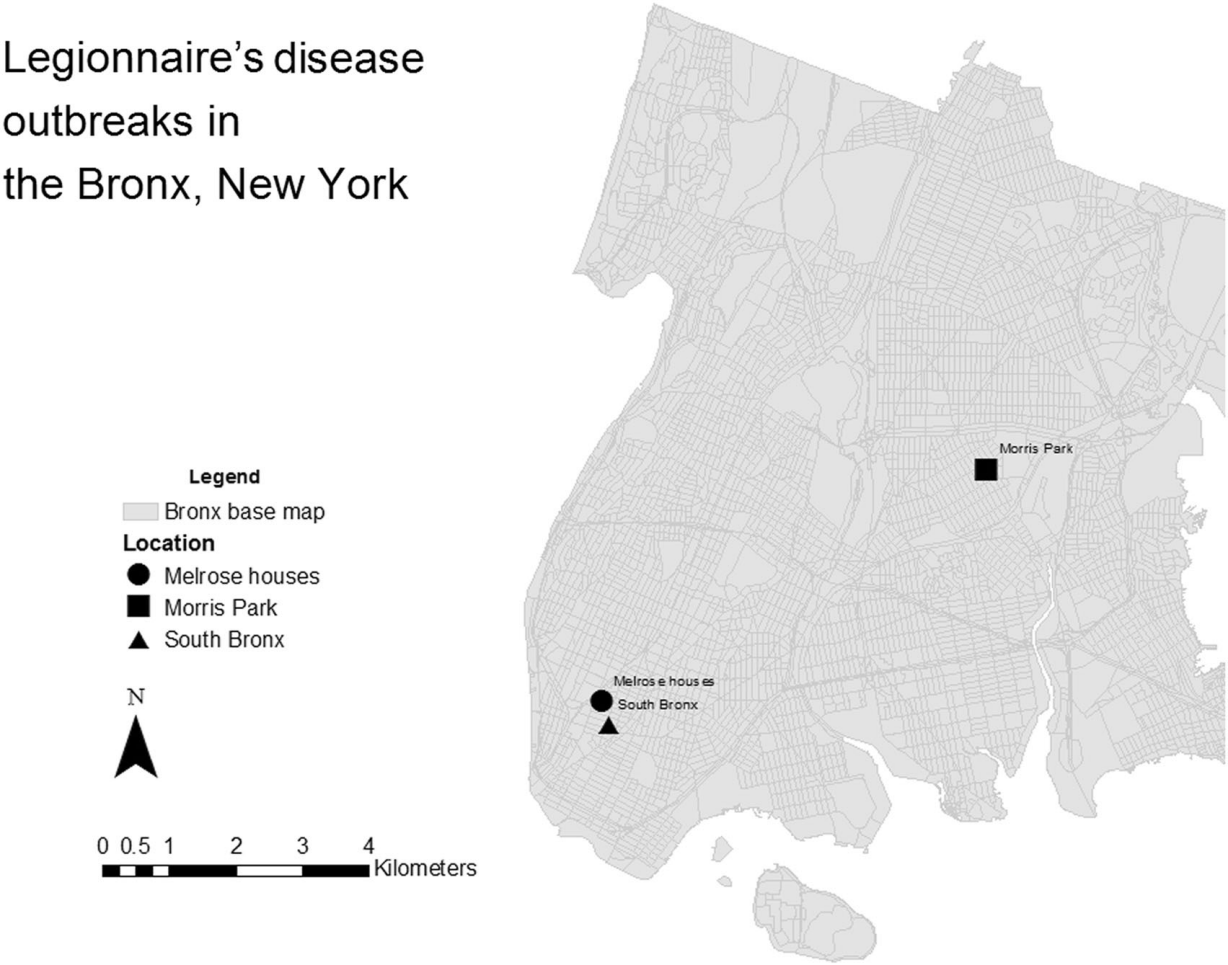
Location of Legionnaires’ disease outbreaks in Bronx, New York City, from 1 March 2015 to 21 September 2015. Outbreak locations are represented by circle (Melrouse houses), square (Morris Park) and triangle (South Bronx) shapes. Grey areas represent the streets within the Bronx district. Outbreak data in the Bronx, NYC was retrieved from the New York City Department of Health and Mental Hygiene and from the Centres for Disease Control and Prevention (CDC) website. Base map data was taken from NYC Department of city planning^17^. Coordinates of outbreak locations retrieved by searching names of locations on Google maps. Maps were created using ArcMap 10.2

Outbreak data in the Bronx, NYC was retrieved from the NYC Department of Health and Mental Hygiene^15^ and from the CDC website^16^. Three outbreaks occurred in the Bronx NYC in a span of 3 months in 2015. The location of the outbreaks in Bronx is shown in Fig. 1.

### Outbreak 1—LD outbreak related to the Opera House in the South Bronx

A large outbreak of LD was reported in South Bronx NYC in July–August 2015. A total of 138 cases and 16 deaths were reported^18^. The median age of cases was 55 years and 62% (86/138) were male. Most of the cases were hospitalized (128/138) and around 77% (107/138) had a pre-existing medical condition^18^. Officials traced that outbreak to a cooling tower at the Opera House Hotel. Strains of *Legionella* bacteria found in the hotel cooling tower were matched with the strain found in Legionnaires’ patients. By October, 21 cooling towers out of the 55 tested have been found to have the *Legionella* in other areas in the South Bronx^18^. The city government mandated that all cooling towers be cleaned within 2 weeks. During the outbreak, NYC health officials mandated that all cooling towers in the South Bronx be disinfected. According to the health department, the water supply was not affected^19^.

### Outbreak 2—LD outbreak in Morris Park, Bronx

Another outbreak of LD started on 21 September 2015 in Morris Park, Bronx, which is located around 4 miles from the Opera House Hotel^19^. This cluster was unrelated to the previous outbreak in the South Bronx, with 13 cases and 1 death reported from 21 to 31 September 2015^20,21^. The age range was 45–75 years and all patients had underlying health conditions. Most patients in this cluster lived or worked in Morris Park. Thirty-five cooling towers in the area were tested for *Legionella* bacteria, and 15 of them were positive^20^. The strain found in the cooling tower at the Bronx Psychiatric Centre matched samples taken from four patients^22^. All towers were cleaned and disinfected immediately. According to a press statement, the NYC drinking water supply was unaffected^21^. According to a news report, when re-tested in September 2015, bacteria were again found in 15 of the 35 *Legionella*-positive cooling towers in Morris Park^23^. According to Legionella Risk Management Inc., this is apparently uncommon as 90% of the time the disinfection is effective and regrowth within a short period of time is rare. This highlights the need for legislation and regulation of cooling towers. Currently, there is no uniform legislation for checking cooling towers in the US, but legislation was introduced in NYC during the South Bronx outbreaks^18^.

### Outbreak 3—LD outbreak in Melrose houses in the South Bronx

**Fig. 2:**
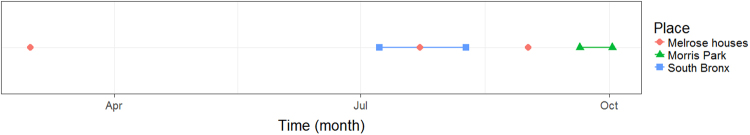
Timeline of Legionnaires’ disease outbreaks in Bronx, New York City, from 1 March 2015 to 21 September 2015. Red circles indicate the date of report of individual cases in Melrose houses. Lines indicate dates of outbreak durations at South Bronx (blue) and Morris Park (green). Outbreak data in the Bronx, NYC was retrieved from the New York City Department of Health and Mental Hygiene and from the Centres for Disease Control and Prevention (CDC) website. Plot was created using R (version 3.1.3)

**Fig. 3:**
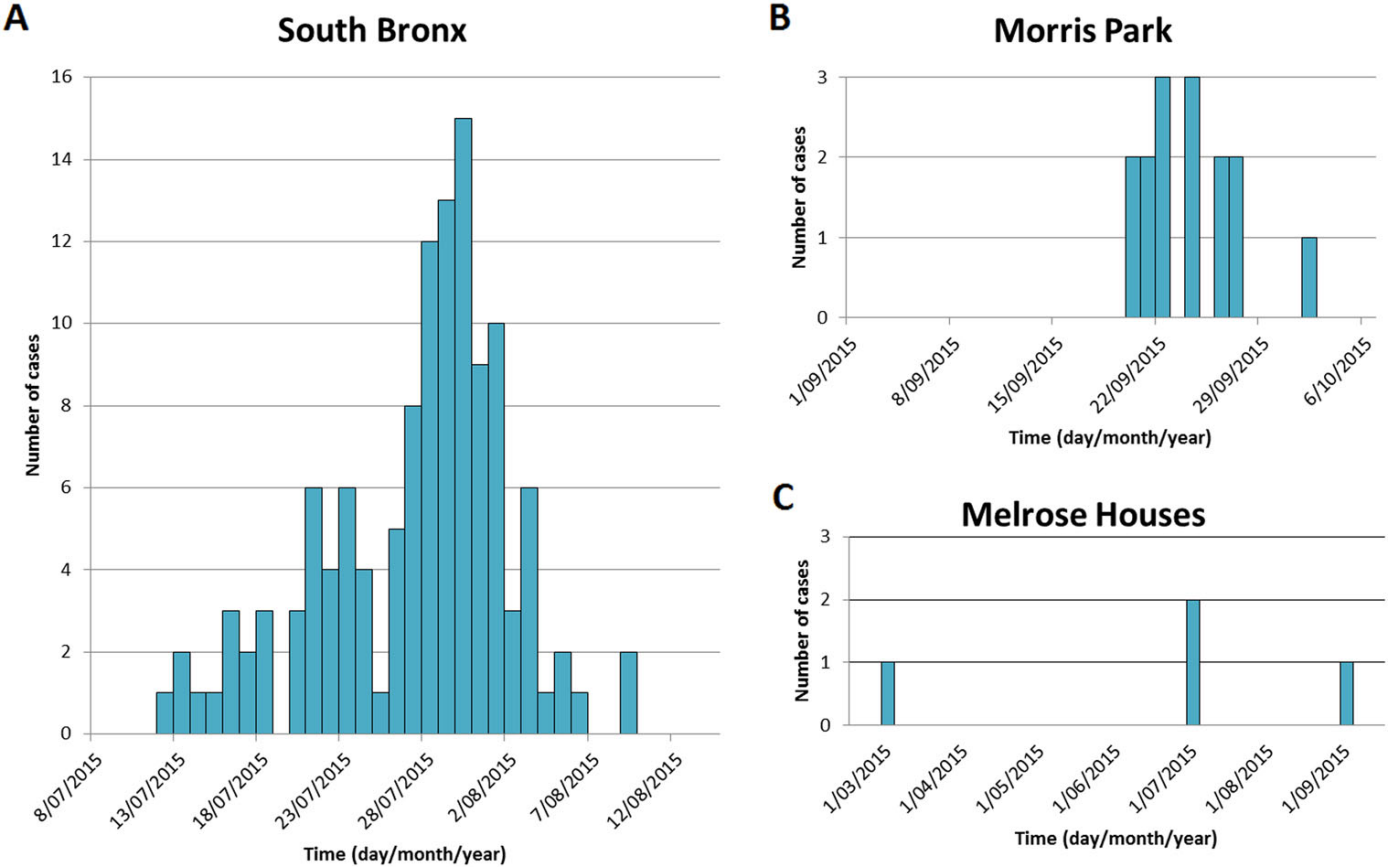
Number of Legionnaires’ disease cases over time in South Bronx, Morris Park and Melrose Houses in New York from 1 March 2015 to 21 September 2015. The first panel **a** shows the number of Legionnaire’s disease (LD) cases in the South Bronx, the top right panel **b** shows Morris Park and the bottom right **c** shows number of cases in Melrose Houses. Outbreak data in the Bronx, NYC was retrieved from the New York City Department of Health and Mental Hygiene and from the Centres for Disease Control and Prevention (CDC) website. Graph was created in Microsoft Excel

A small cluster of LD cases occurred at the Melrose houses in the South Bronx, apparently unrelated to South Bronx and Morris Park outbreaks^20^. Four cases were reported over a period of 6 months—one occurred in the beginning of 2015, two during the South Bronx LD outbreak and one in September 2015^20^. Melrose houses are situated within a one mile radius of the Opera House cooling tower. Cooling towers were not the source in this cluster and bacteria were found in the water distribution system of four of the nine buildings at a Bronx public housing complex. Whether patient strains were matched to environmental strains is unknown. After this outbreak, the Government installed water filters to reduce risk in the Melrose Houses. Figure 2 shows the temporal clustering of outbreaks in the Bronx and Fig. 3 shows outbreak curves.

#### Past outbreaks in New York

**Fig. 4:**
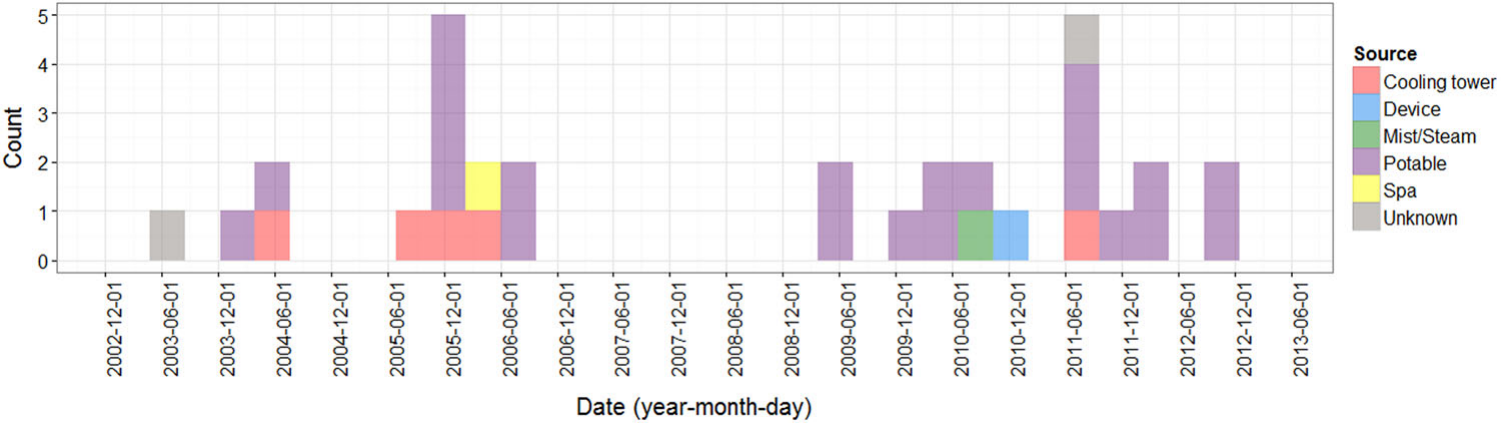
Source and size of outbreaks in New York 2000-2012. coloured bars indicate the number of outbreaks in New York state over time. Bars are coloured according to water source: Cooling tower (red), device (blue), mist/steam (green), potable water (purple), spa (yellow) and unknown (grey). Plot was created using R (version 3.1.3)

A total of 32 outbreaks were reported in NYC from July 2003 to November 2012, and of these, only 5 (16%) were due to cooling towers. Figure 4 shows the source of these different outbreaks, with most being caused by contamination of potable water. A previous study found that 1449 LD cases were reported in NYC from 2002 to 2011. Although most LD cases reported during this period were sporadic, a few healthcare or community-associated clusters were also reported that may or may not have been outbreak related, involving a point source exposure^24^. Of the 155 outbreaks reported in the US from 2000 to 2012, around half (76/155) were due to drinking water and only 7% (11/155) were attributed to cooling towers (7%)^25^.

### Case study 2—multiple LD outbreaks in Sydney

**Fig. 5:**
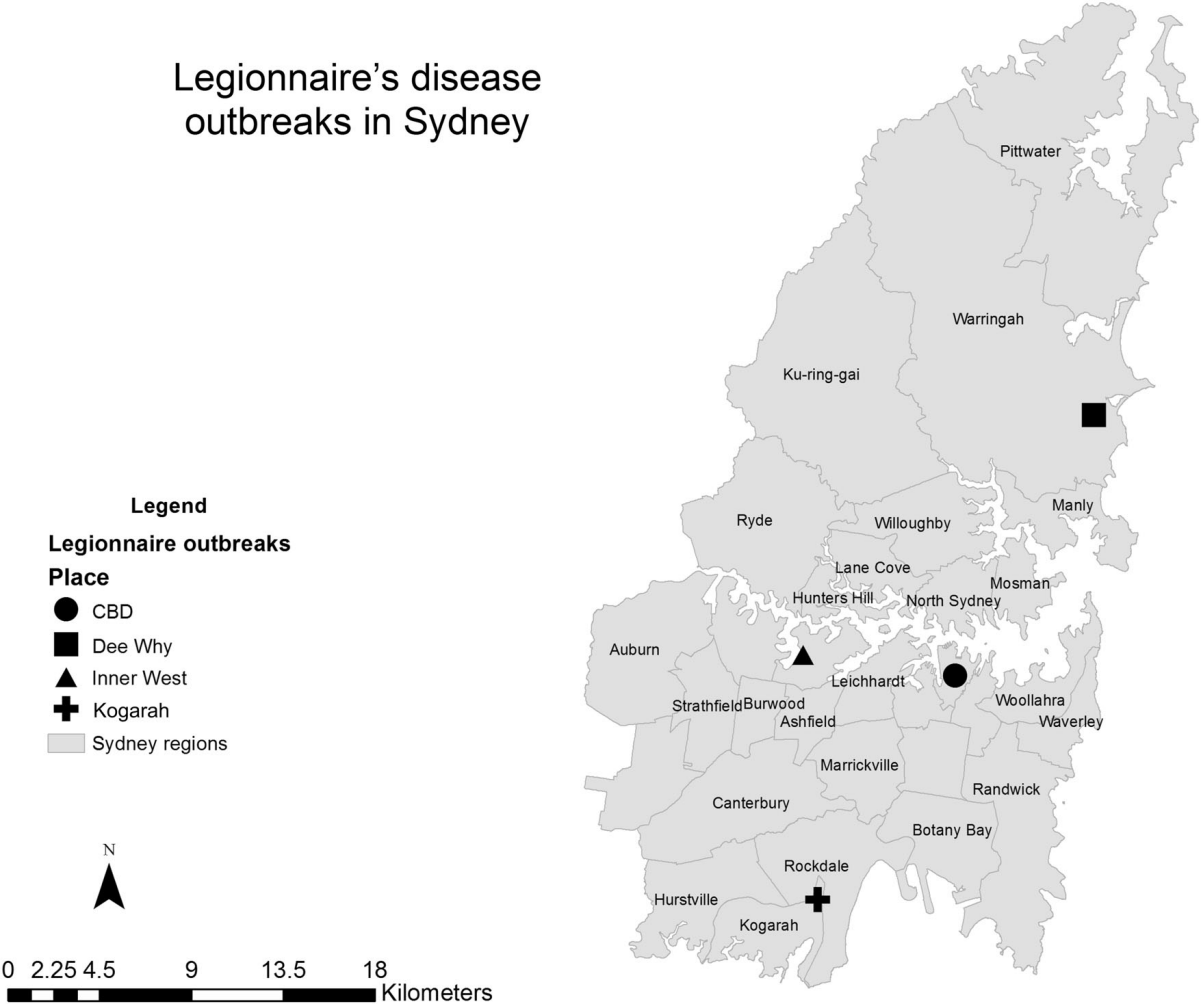
Location of Legionnaries’ disease outbreaks in Sydney, Australia from 27 February 2016 to 29 April 2016. Outbreak locations are represented by circle (CBD district), square (Dee Why), triangle (Inner West) and cross (Kogarah) shapes. Grey areas represent the administrative districts within the Sydney region. Data source of outbreak data: NSW Health^26^. Base map data was taken from the Australian Standard Geographical Classification (ASGC) Digital Boundaries, Australia^14^. Coordinates of outbreak locations retrieved by searching names of locations on Google maps. Maps were created using ArcMap 10.2

**Fig. 6:**
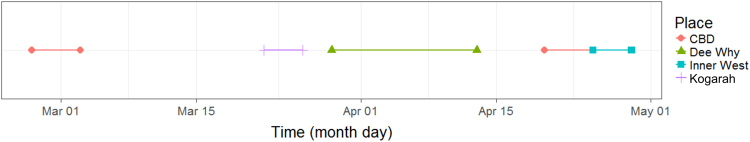
Timeline of Legionnaires’ disease outbreaks in Sydney, Australia, from 27 February 2016 to 29 April 2016. Solid lines indicate dates of outbreak durations in central business district (CBD) locations (red), Kogorah (purple), Dee Why (green) and Inner West (teal) locations. Data source of outbreak data: NSW Health 26. Plot was created using R (version 3.1.3)

Case data (disease onset dates and location of exposure) of multiple LD outbreaks in Sydney in March–May 2016 were obtained from NSW Health^26^. There have been a number of outbreaks of LD identified in Sydney in different locations in 2016^26,27^. The location of the outbreaks in Sydney is shown in Fig. 5 and the timeline of Sydney outbreak is shown in Fig. 6. The first outbreak began in March 2016 (notified to health department on 8 and 9 March 2016), with a further four outbreaks reported between March and June 2016. Using government reports and news article sources, a total of 26 cases associated with the different clusters in the Sydney area were identified: 9 cases in Town Hall in the central business district (CBD) in February/March^26^, 3 cases near Kogarah/Hurstville in March^27,28^, 3 cases in Dee Why in April^29^, 6 cases in the CBD near Wynard station in April/May^26^ and 5 cases in the inner West in May^30^. Two deaths were reported, one in an 80-year-old man in the CBD outbreak in March 2016, and a second in an elderly man associated with the Burwood outbreak in April 2016^26^. The source of these outbreaks is not confirmed; however, several cooling towers tested positive for *L. pneumophilla* serogroup 1 in the CBD area in the initial investigation into the March and May outbreaks^26^. Very high counts of bacteria (>1000 cfu/mL) were isolated from two different cooling towers in the May outbreak. A genetically identical strain was found in both cooling towers and in two of the three culture-positive patients from March outbreak and three of the four culture-positive patients from the May outbreak^26^. There were also two culture-positive cases identified during these outbreaks who had a different genetic sequence and were judged on this basis by the authorities to have acquired their LD elsewhere.

**Fig. 7:**
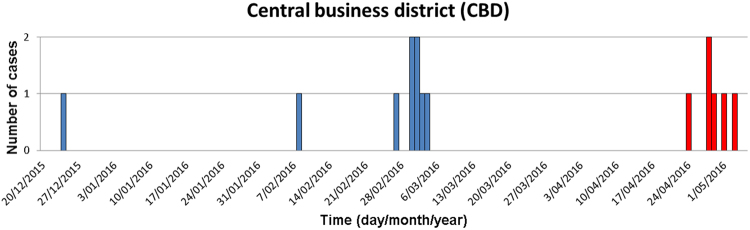
Number of Legionnaires’ disease cases in the central business district of Sydney. Blue bars represent cases in the first outbreak and red bars represent cases during the second outbreak. Data source of outbreak data: NSW Health^26^. Graph was created in Microsoft Excel

Epidemiological curves for two LD outbreaks in CBD Sydney are shown in Fig. 7. In these two outbreaks, two cooling towers harboured the identical strain found in five patients. One-hundred and ninety-nine cooling towers were investigated in both CBD outbreaks^26^. Cooling towers have also been tested in investigations in the other three clusters^27,31,32^. A cooling tower at St George Hospital was identified as a possible source of outbreak in Kogarah^27^. This outbreak was not related to the CBD outbreak^27^. The source of Inner West outbreak could not be identified^30^. NSW Health reports that the sources may never be identified, with cooling towers often being cleaned prior to inspection by the building operators once an outbreak has been publicly announced in the area. An expert working group has been tasked with making recommendations as to whether closer regulation of cleaning and inspecting cooling towers is needed^27^.

**Fig. 8:**
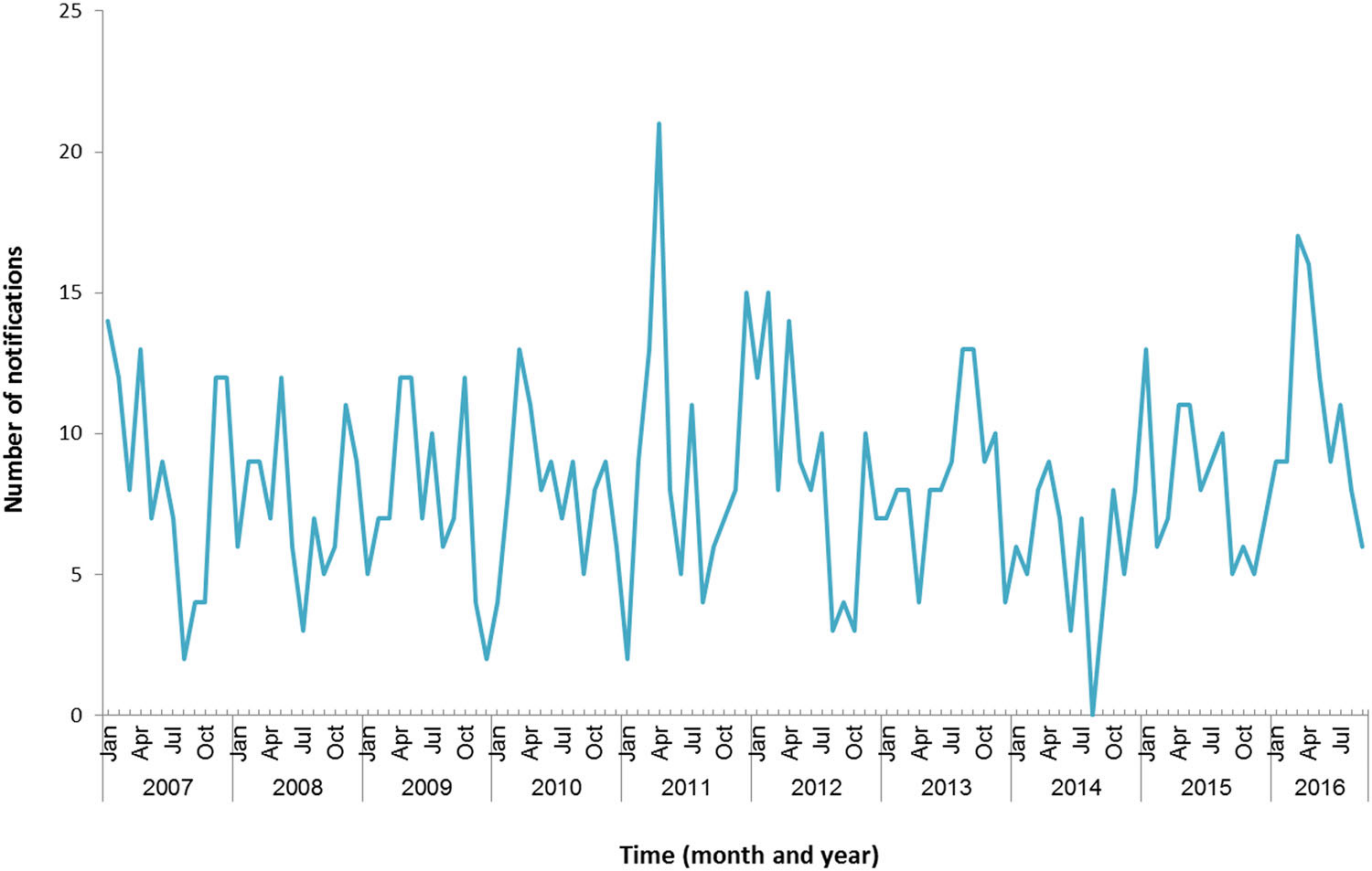
Number of notifications of Legionnaires’ disease in NSW, January 2007–September 2016. Data source: Australia’s notifiable diseases status: Annual report of the National Notifiable Diseases Surveillance System^33^. Graph was created in Microsoft Excel

NSW Health has reported that notifications for the first 4 months of the year (42 notifications) are only slightly higher than the previous 5 years (an average of 37 cases)^33^. Data from the National Notifiable Disease Surveillance System (Fig. 8) shows that numbers of notifications in the first 4 months of the year are not much higher than previous years^33^.

NSW Health reports that there have been improvements in testing in NSW recently, and LD alerts were sent to doctors that may have resulted in improved case ascertainment^34^. The total number of notifications at that time of year is slightly higher than previous years, but the number of outbreaks identified appears higher than previous years. Five outbreaks were identified in Sydney between February and May 2016. There have been a number of previous outbreaks of LD in Sydney since 1990. The majority have been associated with cooling towers.

## Discussion

We describe rolling outbreaks of *Legionella* clustered in time and geography in the Bronx, New York, USA and in Sydney, Australia. Such clustering in time and geography has not commonly been described previously and warrants further critical analysis. The past outbreaks in New York were fairly small compared to the outbreaks in the Bronx, and the majority was linked to hospitals or health facilities. The rate of *Legionella* has increased in New York from 0.8 cases per 100,000 population in 2002 to 2.7 cases per 100,000 in 2009^35^ and this increase could be artefact due to increased testing for *Legionella* or could be a real increase—possibly due to changes in water sanitation, management of cooling towers, seasonal weather changes or other reasons. Climate change and aging populations may have little impact on LD case reports as these are long-term changes. The influence of poverty, underlying patient conditions, seasonal weather changes and technical challenges play a larger role.

The Bronx, the Opera House Hotel and Morris Park outbreaks were linked to cooling towers, but the Melrose outbreak was linked to the water supply. Interestingly, the two cooling tower outbreaks were not related to each other despite being in close proximity.

Similarly in Sydney, it appears unusual to have five outbreaks of *Legionella* within a 4-month period in the Sydney area, with a genetically identical strain found in two different cooling towers and linked to five patients. The finding of more than one contaminated environmental source with an identical genetic sequence is unusual, with most reported outbreaks linked to a single source. Regulation of cooling towers in Sydney is more stringent than NYC (which has no legislation prior to 2015), with mandatory standards for disinfection and random audits of cooling towers^36^. The NSW Ministry of Health have also put in a number of additional public health actions in response to the outbreaks, but it is unclear whether water sources other than cooling towers have been investigated.

Investigation of *Legionella* outbreaks requires matching of environmental samples of *Legionella* with patient samples to determine if the environmental source is likely to have infected the patient. In the Opera House outbreak, the environmental and patient strains were matched^18^; however, no data have been reported on matching to date on the remaining outbreaks in the Bronx and Sydney. Beyond this, the genetic epidemiology of the outbreak *Legionella* isolates need to be studied further to assess their phylogenetic relatedness at each site. A review of sporadic, epidemic and environmental *Legionella* isolates from 1982 to 2014 by the US CDC showed that *Legionella* pneumophila serogroup 1 (Lp1, also the causative strain in the Bronx outbreaks) was the most common and that outbreak and sporadic strains were more similar to each other than to environmental isolates^37^. The most frequent sequence type was ST1, and they hypothesised that this may have increased pathogenicity^37^. A study in Europe shows recent unusual and surprising phylogenetic diversity in *Legionella* strains^38^. They describe recently emerged and globally dispersed clones, which they explain as adaptation to new built environments.

*Legionella* is a notifiable disease in both US and Australia. In the US, cases are monitored through Nationally Notifiable Diseases Surveillance System (NNDSS) and Supplemental LD Surveillance System (SLDSS)^11^. In addition to routine surveillance, outbreaks are reported through the Waterborne Disease Outbreak Surveillance System^10^. In Australia, the NNDSS collects LD data from states and territories^39^. The reporting standards are generally good in both countries; however, there is likely underestimation, especially of sporadic cases, as many *Legionella* cases may be asymptomatic or may not be notified due to mild illness. Cases requiring hospitalization may not be tested for *Legionella* and treated with broad spectrum antibiotics. In the Sydney outbreak, 61 possible LD cases identified through surveillance for pneumonia were tested retrospectively following the outbreak, and three were positive^40^, indicating that around 5% of undiagnosed pneumonia may be due to LD.

Cooling towers are more commonly the source of *Legionella* in Australia than in the US, whereas potable water supply is a more common source in US outbreaks, as in the Melrose housing outbreak^11^. A range of factors need to be considered in understanding and mitigating these epidemics, including surveillance systems and regulation of cooling towers and water systems. Climate variation is also a factor—warmer weather facilitates growth of *Legionella* in biofilms—and is associated with outbreaks. This may explain rolling outbreaks, with unseasonably warm weather in Sydney in 2016, extending beyond the summer months^41^. The Bronx outbreaks also spanned the summer, with temperatures slightly warmer compared to the preceding year^42^.

Routine monitoring and testing of cooling towers is not mandated in the US. A *Legionella* control standard (188–2015) has been developed by the American Society of Heating, Refrigerating, and Air-Conditioning Engineers (ASHRAE®) which is a non-profit organization^43^. The ASHRAE standard is a voluntary consensus Standard in form of a copyrighted document that is not freely available and has to be purchased from ASHRAE website^43^. After two outbreaks in Bronx, NYC was the first major city in the US to pass new legislation to mandate regular testing of cooling towers for *Legionella* bacteria^44^ (NYC local law 77 of 2015. NYC Adm Code, title 28, article 317). In Australia, states mandate regular, routine testing of cooling towers^45^.

Australia has comparatively stringent laws including state-based Public Health Acts^40^. Under the NSW State Public Health Regulations of 2012, for example, regulation and testing of cooling towers is mandated^45^. Installation, operating and maintenance requirements have been outlined in reference policy documents. In a 2011 compliance survey among 276 random cooling tower samples, only 5% had total *Legionella* of >10 cfu/mL^46^. Other jurisdictions in Australia also have regular audits of cooling towers.

Following identification of the clusters, the NSW Ministry of Health has put in place a number of public health actions^40^. Water service treatment providers have been required to send in all cooling tower documentation regarding cooling towers inspected since November 2015. All cooling towers that have tested positive in the investigations are further investigated. Business owners have also been notified by the Sydney council to clean all cooling systems within a set timeframe or face inspection and a fine^47^.

Drinking water is the most common source of LD in the US. A 2015 report by the US Environmental Protection Agency found that 67% of drinking water storage tanks sampled contained *Legionella*. Another EPA study published in 2014 found *Legionella* in 47% of cold water taps sampled nationwide. Interestingly, the Southwest US has relatively few cases of LD while the Northeast has roughly twice the national average^48^. According to the CDC, in the South and Southwest US the potable distribution system tends to approach 1 parts per million (ppm) chlorine while in the Northeast and central North it is <0.5 ppm. Chlorination standards for water in Australia suggest that the chlorine level should be between 0.2 and 0.5 mg/L^49^. In contrast, except for a few hospital outbreaks, LD outbreaks have not been associated with the water supply in Australia^50,51^.

According to a report from a meeting of professionals in the cooling technology industry, all cooling towers in NYC were disinfected at the end of first outbreak in South Bronx^20^ and emergence of another outbreak in Morris Park raised questions around the source of this outbreak. It is possible the general climatic conditions favoured *Legionella* growth, and some cooling towers in Morris Park were not disinfected properly. However, cooling towers are not the main source of *Legionella* outbreaks in the US. A recent report suggests that the measures taken by NYC were not adequate because they did not address an important source of *Legionella* exposure, the drinking water^20^. In the US, there have recently been two examples of neighbourhood-scale or city-scale outbreaks, which were linked directly to the potable water distribution system. In New Jersey, the drinking water was investigated because there were no cooling towers in the area that could explain the outbreak^52^. The second example is Flint Michigan, where corrosion in the distribution system led to city-wide proliferation of *Legionella*, particularly at the ends of the main water pipes where concentrations of disinfectant had progressively decreased before entering plumbing in homes^53^. It is interesting to note in Flint that infections were caused by multiple strains of *Legionella*, suggesting a system-wide problem of maintenance.

A limitation of this study was that most information on LD cases and epidemiological investigations were taken from public reports and news items, rather than official health department records. There is a possibility that some public information may be incorrect or incomplete.

In summary, we present unusual geographically and temporally clustered *Legionella* outbreaks in two cities, for which an explanation is not clear. For example, in relation to the Bronx, the Opera House Hotel and Morris Park outbreaks, it is unusual for so many cooling towers to be contaminated at one time. In addition in relation to the Sydney outbreaks, it is unusual to have five outbreaks occurring in the same city within 4 months and unusual to find more than one contaminated environmental source with an identical genetic sequence. In contrast to the Flint Michigan outbreak^53^, where a systematic problem with the water supply was identified that explained ongoing LD cases from a single source, these rolling outbreaks stand out as different from past *Legionella* outbreaks. This is a concerning trend that requires further investigation and mitigation.
